# Case report: Ultrahypofractionated palliative breast radiotherapy for a fungating invasive mammary carcinoma

**DOI:** 10.3389/fonc.2023.1171444

**Published:** 2023-06-07

**Authors:** Yejun Hong, Nancy Nixon, Jeffrey Q. Cao, Sangjune Laurence Lee

**Affiliations:** ^1^ Cumming School of Medicine, University of Calgary, Calgary, AB, Canada; ^2^ Division of Medical Oncology, University of Calgary, Calgary, AB, Canada; ^3^^†^ Division of Radiation Oncology, University of Calgary, Calgary, AB, Canada

**Keywords:** case report, breast cancer, ultrahypofractionated, radiotherapy, palliative, radiation complications

## Abstract

Palliative radiotherapy for symptomatic and intact breast tumors must balance convenience, efficacy, and risk of acute toxicity. This case report presents a patient with metastatic breast cancer and an intact fungating primary tumor. She was treated with an ultrahypofractionated radiation therapy, 26 Gy in 5 consecutive daily fractions, with sequential palliative chemotherapy. This resulted in a minimal toxicity profile and significant reduction of tumor burden and symptoms.

## Introduction

Invasive breast cancer affects one in eight women and is the second most common cause of death from cancer in the female population (1). There are well-established breast cancer screening guidelines, which includes a biennial mammogram for women starting as early as 40 and typically up to age 74, or annual breast ultrasound or MRI for high-risk individuals between ages 30 and 69. Unfortunately, with the recent COVID-19 pandemic, there has been a significant delay in screening breast imaging due to government mandated measures designed to mitigate the spread of the virus (2). The pandemic has contributed to an increased incidence of advanced stage breast cancer at initial presentation, especially in marginalized populations with lower income and complex medical comorbidities (3). This case report explores the effectiveness of the ultrahypofractionated radiation dose and fractionation schedule of 26 Gy in 5 consecutive daily fractions as an alternative to common palliative dose and fractionation schedules for a patient with an intact, fungating, locally advanced breast cancer in the setting of metastatic disease.

## Case description

An 83-year-old female presented to the local emergency department with a six-month history of an enlarging non-tender lump in her left breast. The patient reported a two-month history of white purulent discharge, bleeding from the mass, and 1-month history of constitutional symptoms. She had an ECOG performance status of 2. The patient had no prior mammograms or cancer screening. Prior to her admission, the patient was living in a seniors’ living facility and was independent of all her activities of daily living. In terms of comorbidities, the patient was taking apixaban due to a pulmonary embolism diagnosed 1 year prior.

**Figure 1:**
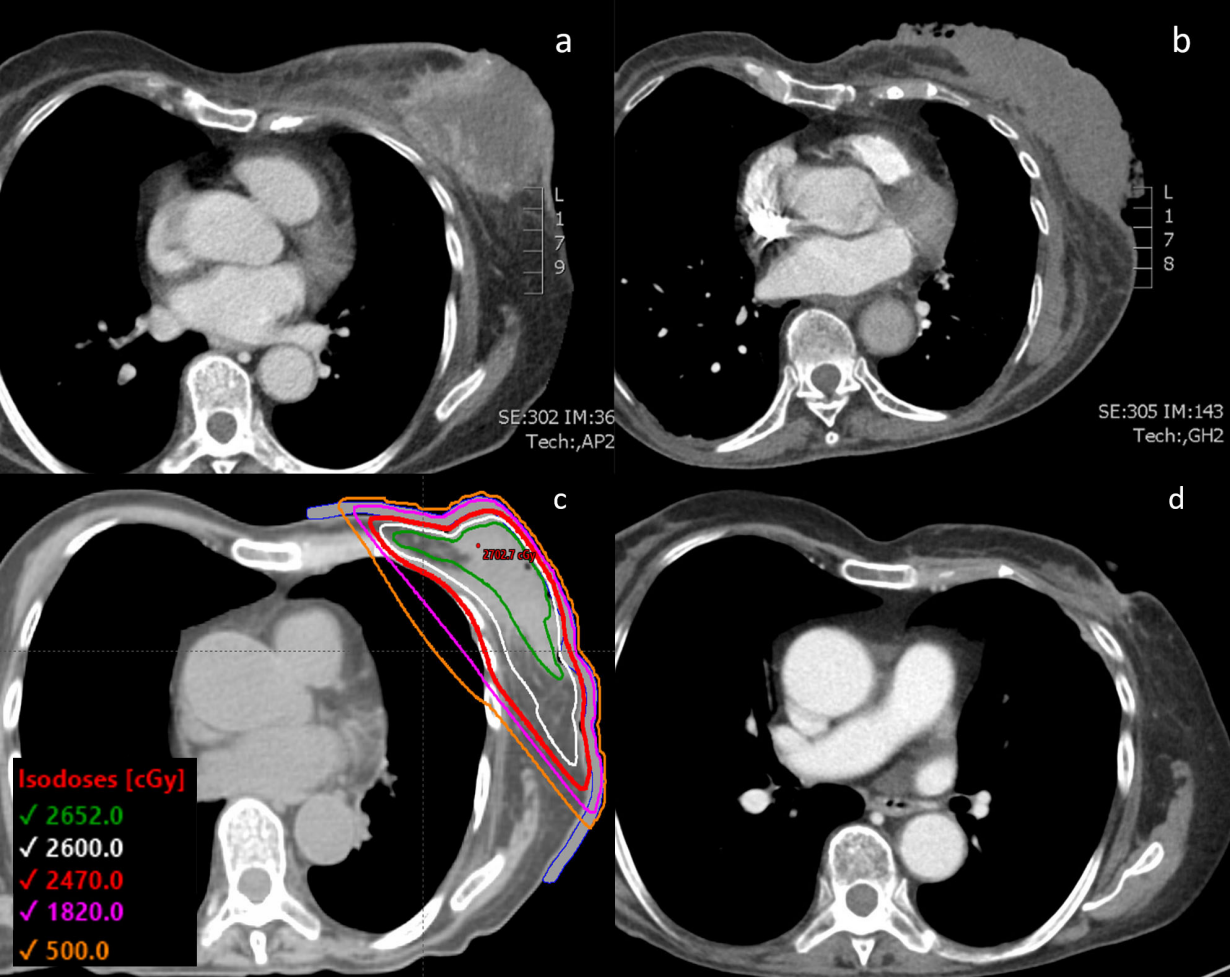
**(A)** CT chest of the patient with the 12 cm diameter left-sided fungating breast mass shown at the time of initial diagnosis. **(B)** Tumor visualized after progressing through initial line of paclitaxel chemotherapy and right before initiating FAST-Forward radiation therapy. **(C)** Radiotherapy plan with prescribed dose of 26 Gy in 5 fractions with a 0.5 cm bolus. **(D)** CT chest showing the 1.8 cm tumor 3.5 months after completion of radiation therapy and starting doxorubicin chemotherapy.

Breast examination revealed a large, purulent, and ulcerated breast mass of 10 cm x 12 cm at the 1 o’clock position of the left breast. Another smaller and purulent mass was found at 4 o’clock position on the same side. The left breast was erythematous, but with no peau d’orange or nipple inversion. There was a small, mobile, palpable left axillary lymph node. Chest CT was obtained, which showed the large fungating left breast mass (**Figure 1A**), abnormally enhancing axillary and retropectoral lymph nodes, and multiple non-specific pulmonary nodules. Further staging examinations included a nuclear bone scan which revealed a focus of increased activity in the sacrum, consistent with metastases. Biopsy of the left breast mass showed an estrogen receptor negative, progesterone receptor negative, and HER2-negative (i.e., triple-negative) invasive mammary carcinoma, grade 2. The patient was staged with cT4bN1M1 disease.

The patient agreed to initiate palliative chemotherapy. She was treated with a 3-week cycle of weekly paclitaxel, with a 1-week break per cycle (**Table 1**). After 3 cycles of paclitaxel, she was found to have progression in the breast and lungs, and thus was switched to a 3-week cycle of weekly doxorubicin with a 1-week break per cycle. During this chemotherapy, she experienced a hypersensitivity reaction that resulted in a delay in treatment. With resumption of therapy, she also developed worsening purulent drainage from her left breast mass, indicating infection. After 3 cycles of doxorubicin, her follow-up chest CT revealed an increase in size of the left breast mass as well as of the pulmonary nodules (**Figure 1B**). Doxorubicin was stopped and the patient underwent ultrahypofractionated radiation therapy to the whole left breast with 26 Gy over 5 fractions (**Figure 1C**). The patient was simulated in the supine position with the arms over the head in a wing-board with deep-inspiration breath hold using real-time position management. A 3-dimensional conformal radiation therapy plan was used covering the whole breast from the inferior aspect of the clavicle superiorly, mid-sternum medially, 2 cm below the inframammary fold inferiorly, and mid-axillary line laterally. Tangent fields with half beam blocks and 6 MV energy with a 5 mm bolus covering the skin were used. The FAST-Forward protocol dose objectives and constraints were all met (4). The left breast V95% was 98.4%, heart mean dose was 0.35 Gy, coronary artery mean was 0.99 Gy, left lung V8 Gy was 8.42% and V12.5 Gy was 6.8%, and the maximum dose was 104.7% of the prescription dose. Daily KV orthogonal pair imaging was used to ensure proper positioning.

**Table 1:** Timeline of episode of care.

Date relative to initial presentation	Event
6 months prior	First noticed golf ball sized mass in left breast
Initial presentation	Presented to hospital with grapefruit sized fungating left breast mass and bone metastases
1 month post	Initiated palliative paclitaxel. 3 cycles delivered
4 months post	Initiated palliative doxorubicin. 3 cycles delivered.
7 months post	Palliative whole breast radiotherapy, 26 Gy in 5 fractions. Doxorubicin restarted. 3 further cycles of doxorubicin delivered.
11 months post	CT of chest showed that the breast mass had shrunk to 1.8 cm in diameter. Patient continued on palliative doxorubicin.
13 months post	At last follow up, patient had no complications from radiotherapy or worsening symptoms

The patient was assessed 1 month after completion of radiotherapy by the radiation oncology team, after which her care was continued with medical oncology. Treatment response was evaluated through physical exam and imaging. The patient experienced mild radiation dermatitis from the radiation therapy, and subsequently, re-epithelization over the breast mass eliminated the need for further wound dressings. Two weeks after completing radiotherapy, the patient restarted palliative doxorubicin. 3 cycles of doxorubicin were delivered after the completion of radiotherapy, at which point the patient underwent a restaging CT. This chest CT taken 3.5 months after completion of the ultrahypofractionated radiation therapy showed that the mass had significantly reduced and measured 0.8 cm by 1.8 cm (**Figure 1D**). The 6-month follow-up showed that the patient remained without complications from the radiotherapy or worsening symptoms.

## Discussion

Palliative radiotherapy must provide effective cancer control and symptom relief, with minimal burden on quality of life. Local control and relief are especially important in patients with metastatic breast cancer, who have a prognosis extending to a range of five years (5). Conventional palliative dose fractionation schedules, however, are either long and inconvenient or short and associated with poor local control (6).

The FAST-Forward study proposed a new, more efficient fractionation schedule that delivers 26 Gy over 5 fractions of ultrahypofractionated radiotherapy in the adjuvant setting. The study demonstrated non-inferiority of this novel dose and fractionation scheme compared to moderately hypofractionated radiotherapy in terms of local cancer control and cosmetic outcomes (4). The FAST-Forward study showed minimal complications, with moderate to brisk erythema in 30% of the patients and skin desquamation in 11% (7). This schedule is used routinely in many parts of Canada in the adjuvant setting.

Using the α/β ratio of 3.7 Gy for breast tissues, the equivalent total dose in 2-Gy fractions (EQD2) for 26 Gy over 5 fraction is 40.6 Gy, which is very similar to the EQD2 of 44.7 Gy for 40 Gy over 15 fractions used in adjuvant radiotherapy (4). On the other hand, commonly used short palliative radiotherapy dose and fractionation schedules such as 8 Gy in 1 fraction has an EQD2 of 16.4 Gy, and 20 Gy in 5 fractions has an EQD2 of 27 Gy. Conventionally fractionated palliative radiotherapy (ranging from 39 Gy in 13 fractions [EQD2 of 45.84 Gy] to 50 Gy in 25 fractions [EQD2 of 50 Gy]) to intact breast tumors require re-irradiation in 18% of patients with a mean time to re-irradiation of 16 months. 8 Gy delivered in a single fraction requires re-irradiation in 44% of cases, with a mean time to re-irradiation of 3 months (8). The FAST-Forward treatment dose and fractionation can deliver higher doses than the more commonly used short palliative dose and fractionation schedules, while achieving local control rates similar to longer conventional fractionation schedules.

In addition to improved local control, there are many benefits to choosing shorter dose-fractionation schedule. Longer fractionation schedules have a higher risk of premature treatment termination. More frequent visits over longer periods of time are a significant burden for patients and their families with limited options for transportation and financial support. At a time of a public health emergency like the COVID-19 pandemic, longer radiotherapy schedule raises the risk of iatrogenic infections, as well as straining the healthcare resources (9).

This case report illustrates the efficacy of the ultrahypofractionated dose-fractionation schedule for the palliation of intact symptomatic breast tumors while minimizing treatment burden. Given that patients with metastatic breast cancer may have a prognosis of several years, delivering a higher EQD2 may provide optimal local control and symptom control.

## Patient perspective

Our patient who agreed to be part of the case report commented following regarding her radiation treatment: “I did experience some itchiness of the skin for about a month after radiation, but I didn’t need any medication for this. I didn’t have much change in my energy levels. The tumor shrank down and became much more manageable, and I was able to proceed with chemotherapy. The radiation therapy staff were very friendly”.

## Data availability statement

The original contributions presented in the study are included in the article/supplementary material. Further inquiries can be directed to the corresponding author.

## Ethics statement

Ethical review and approval was not required for the study on human participants in accordance with the local legislation and institutional requirements. The patients/participants provided their written informed consent to participate in this study. Written informed consent was obtained from the individual(s) for the publication of any potentially identifiable images or data included in this article.

## Author contributions

YH: organized the case and wrote manuscript; SL: administered radiation therapy for patient, supervised writing of manuscript; JC: suggestion for edits a senior radiation oncologist; NN: administered chemotherapy for patient, suggestion for edits from perspective of medical oncologist. All authors contributed to the article and approved the submitted version.
